# Supplementary material for: Antioxidant and Cytoprotective Properties of Cyanobacteria: Potential for Biotechnological Applications
Source: Toxins (Basel). 2020 Aug 26;12(9):548. doi: 10.3390/toxins12090548 (PMC7551995; doi:10.3390/toxins12090548)
Supplement: Supplementary file 1 [file toxins-12-00548-s001.pdf]

Supplementary Materials: Antioxidant and cytoprotective properties of cyanobacteria: potential for biotechnological applications

Adriana Guerreiro, Mariana A. Andrade, Carina Menezes, Fernanda Vilarinho and Elsa Dias

Table S1. Cell volume and antioxidant content expressed "per cell" and "per cell volume" of cyanobacterial strains.

Cyanobacterial Strains	Cell Volume (µm³)	Solvent	DPPH Inhibition		Phenolic Compounds		Flavonoid Compounds	
			TE pg/cell	TE pg/µm³	GAE fg/cell	GAE fg /µm³	QE fg /cell	QE fg /µm³
LMECYA 173	1.48	Methanol	0.064 ± 0.003	0.042 ± 0.002	2.45 ± 0.04	1.63 ± 0.02	24.0 ± 0.7	16.0 ± 0.5
		Ethanol	0.013 ± 0.002	0.009 ± 0.002	2.91 ± 0.03	1.94 ± 0.02	15.34 ± 0.1	10.24 ± 0.07
LMECYA 257	8.19	Methanol	1.25 ± 0.08	0.156 ± 0.009	74 ± 1	9.2 ± 0.1	489 ± 4	61.2 ± 0.5
		Ethanol	0.012 ± 0.000	0.001 ± 0.007	52 ± 1	6.49 ± 0.09	219 ± 5	27.3 ± 0.6
LMECYA 180	10.08	Methanol	3.4 ± 0.2	0.34 ± 0.02	200 ± 2	20.0 ± 0.2	571 ± 19	57 ± 2
		Ethanol	0.66 ± 0.04	0.066 ± 0.004	240.3 ± 0.4	24.03 ± 0.05	880 ± 9	88 ± 1
LEGE 06224	11.33	Methanol	2.2 ± 0.3	0.20 ± 0.02	124.5 ± 0.6	11.3 ± 0.05	1002 ± 20	91 ± 2
		Ethanol	2.20 ± 0.03	0.200 ± 0.002	197 ± 5	17.9 ± 0.5	912 ± 22	83 ± 2
LMECYA 009	12.69	Methanol	0.207 ± 0.001	0.0159 ±0.0001	34.6 ± 0.3	2.66 ± 0.02	189 ± 2	14.5 ± 0.5
		Ethanol	0.04 ± 0.02	0.003 ± 0.001	52 ± 2	4.0 ± 0.1	308 ± 3	23.7 ± 0.2
LMECYA 127	22.26	Methanol	2.0 ± 0.2	0.09 ± 0.01	165 ± 7	7.5 ± 0.3	733 ± 8	33.3 ± 0.3
		Ethanol	0.5 ± 0.1	0.021 ± 0.004	102 ± 1	4.65 ± 0.07	564 ± 3	25.6 ± 0.2
LMECYA 088	25.99	Methanol	0.10 ± 0.01	0.0039 ± 0.0004	11.5 ± 0.3	0.44 ± 0.01	37 ± 1	1.41 ± 0.04
		Ethanol	0.042 ± 0.007	0.0016 ± 0.0002	35.3 ± 1.2	1.36 ± 0.05	258 ± 1	9.92 ± 0.05
LMECYA 291	36.88	Methanol	13.1 ± 1.1	0.354 ± 0.03	1099 ± 17	29.7 ± 0.5	3744 ± 119	101 ± 3
		Ethanol	4.312 ± 0.001	0.12 ± 0.06	964 ± 10	26.1 ± 0.3	3373 ± 99	91 ± 3
